# Computationally predicted gene regulatory networks in molluscan biomineralization identify extracellular matrix production and ion transportation pathways

**DOI:** 10.1093/bioinformatics/btz754

**Published:** 2019-10-16

**Authors:** Victoria A Sleight, Philipp Antczak, Francesco Falciani, Melody S Clark

**Affiliations:** 1 Department of Zoology, University of Cambridge, Cambridge, UK; 2 Biodiversity, Evolution and Adaptation Team, British Antarctic Survey, Cambridge, UK; 3 Department of Functional and Comparative Genomics, Institute of Integrative Biology, University of Liverpool, Liverpool, UK

## Abstract

**Motivation:**

The molecular processes regulating molluscan shell production remain relatively uncharacterized, despite the clear evolutionary and societal importance of biomineralization.

**Results:**

Here we built the first computationally predicted gene regulatory network (GRN) for molluscan biomineralization using Antarctic clam (*Laternula elliptica*) mantle gene expression data produced over an age-categorized shell damage-repair time-course. We used previously published *in vivo in situ* hybridization expression data to ground truth gene interactions predicted by the GRN and show that candidate biomineralization genes from different shell layers, and hence microstructures, were connected in unique modules. We characterized two biomineralization modules of the GRN and hypothesize that one module is responsible for translating the extracellular proteins required for growing, repairing or remodelling the nacreous shell layer, whereas the second module orchestrates the transport of both ions and proteins to the shell secretion site, which are required during normal shell growth, and repair. Our findings demonstrate that unbiased computational methods are particularly valuable for studying fundamental biological processes and gene interactions in non-model species where rich sources of gene expression data exist, but annotation rates are poor and the ability to carry out true functional tests are still lacking.

**Availability and implementation:**

The raw RNA-Seq data is freely available for download from NCBI SRA (Accession: PRJNA398984), the assembled and annotated transcriptome can be viewed and downloaded from molluscDB (ensembl.molluscdb.org) and in addition, the assembled transcripts, reconstructed GRN, modules and detailed annotations are all available as Supplementary Files.

**Supplementary information:**

Supplementary data are available at *Bioinformatics* online.

## 1 Introduction

The evolution of biomineralization in the late Precambrian corresponded with a huge expansion in morphological diversity. Representatives of all Kingdoms form biominerals, they are a substantial feature of life as we know it. One phylum which owes its success to biomineralized shell is the Mollusca (Vermeij, 2005). With over 85 000 extant species, molluscs are the second most speciose animal phyla. They are essential components in worldwide ecosystem functioning, an important source of protein for growing human demand and show biomimicry potential for the development of strong, low-energy, materials from vastly abundant soluble calcium carbonate (Finnemore *et al.*, 2012). In light of the clear social and economic importance of biominerals and biomineralizing organisms, ocean acidification is a cause of concern (Gazeau *et al.*, 2013), and environmental scientists are trying to predict the fate of calcifiers under future ocean acidification scenarios. There is therefore, a clear requirement to understand fundamental mechanisms of molluscan biomineralization, yet to date, such mechanisms are poorly characterized.

Molluscan shells form through a controlled biological process producing a composite biomaterial containing 95–99% calcium carbonate (CaCO_3_) and 1–5% organic matrix. In order to build their shells, molluscs transport minerals, proteins, glycoproteins, lipids and carbohydrates across the mantle to the extrapallial space, where mineral components are laid down as organized crystals onto an organic matrix. Over the last decade, mantle transcriptomes and shell proteomes have been described for many mollusc species (Clark *et al.*, 2010; Jackson *et al.*, 2006; Marie *et al.*, 2010, 2013), in addition to RNA-Seq shell damage-repair experiments (Huning *et al.*, 2016; Sleight *et al.*, 2015). A handful of candidate biomineralization genes have been identified and further characterized with a combination of qPCR, *in situ* hybridizations and protein immune-detection (Fang *et al.*, 2012; Marie *et al.*, 2012; Sleight *et al.*, 2016). Annotation rates vary considerably between mollusc species from around 32–34% in the Antarctic clam and the blue mussel (*Mytilus edulis)* to 87% in the Pacific oyster (Sleight *et al.*, 2015; Yarra *et al.*, 2016), which often constrains research efforts to previously characterized, annotated sequences. Much of the molluscan shell molecular tool kit remains poorly described and many of these ‘unknown’ genes will play important roles in the biomineralization process. There are currently no data available on gene regulatory networks (GRN) controlling biomineralization in molluscs and tools are urgently required to extend analyses beyond a small set of candidate genes and proteins.

GRNs can be inferred from observational data, including transcriptomes; can identify regulators of complex biological processes and, using a guilt-by-association criteria, can formulate hypotheses on the function of non-annotated genes. To gain insight into the transcriptional regulation of molluscan biomineralization we used a global gene expression profiling dataset and an information theory approach to reverse engineer underlying GRNs.

## 2 Materials and methods

### 2.1 Experimental design

**Fig. 1.**
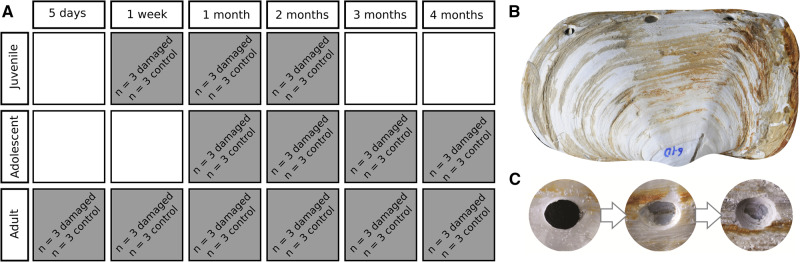
Experimental design of the perturbation time-course. (**A**) Schematic representation of experimental design, 78 individual animals sampled over six time points post-perturbation, three age categories and two treatments (damaged shell and undamaged control). (**B**) Example of shell damage treatment to adult shell. (**C**) Example of repair over time [left to right: 1, 2 and 3 months post-perturbation, modified from Sleight *et al.* (2015)]

In order to induce biomineralization, time-course shell damage-repair experiments were conducted on three age categories of Antarctic clam *Laternula elliptica* (juvenile = 1–2 years, adolescent = 3–5 years, adult = > 6 years) using a previously described protocol for shell damage and mantle tissue collection (Sleight *et al.*, 2015). In order to use data to predict gene interactions experiments included perturbation (shell-damage) versus control, over a time course (experimental design summarized in Fig. 1).]

### 2.2 Data analysis

Summarized here is the outline of the analysis pipeline, for full data analysis methods please see Supplementary Material. RNA-Seq libraries were *de novo* assembled and transcript abundance was estimated using alignment-based quantification. SOTA was first used to cluster transcripts based on expression profiles (Herrero *et al.*, 2001), and subsequent clusters were input into ARACNe to reverse engineer the GRN (Margolin *et al.*, 2006). Previously characterized molecular markers for biomineralization were used to ground-truth the GRN. Time-dependent damage-response genes were identified using edgeR and mapped onto the GRN (Robinson *et al.* 2010). Biomineralization modules were further investigated using enrichment analysis and an *in silico* screening criteria was applied to identify priority candidates in the regulation of molluscan biomineralization.

## 3 Results and discussion

### 3.1 First computationally predicted mantle-specific GRN resource for molluscs

**Fig. 2.**
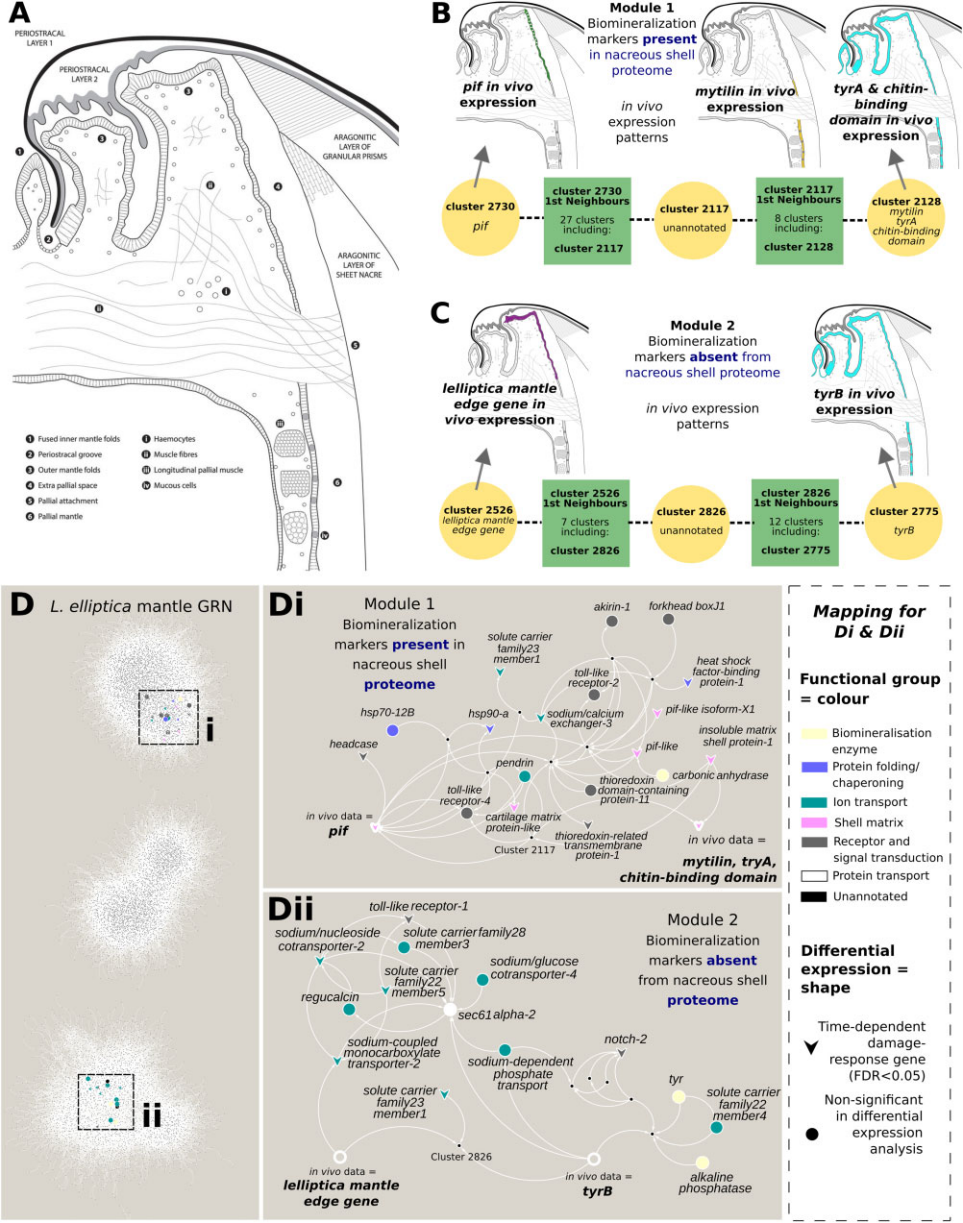
Biomineralization modules of the GRN ground-truthed with previously published *in situ* hybridization *in vivo* expression data. (**A**) Schematic of *L.elliptica* mantle anatomy and shell [modified from Sleight *et al.* (2016)]. (**B**) Module 1 regulates the secretion of extracellular matrix proteins required for biomineralization of the nacreous shell layer. (**C**) Module 2 regulates the transportation of ions and proteins required for biomineralization. (**D**) Overview of the GRN with priority gene candidates from modules 1 (i) and 2 (ii) and their closest connections, colour and shape mapping functional group and differential expression status, respectively. More information on all of the transcripts involved in these modules, their annotations, as well as the sequence data for the transcripts, is available in Supplementary Files S3 and S4 for module 1 and 2 respectively

We present the first computationally predicted GRN for molluscan biomineralization constructed from age-categorized damage-repair mantle gene expression data (Supplementary File S2, Fig. 2, Supplementary Fig. S1). Owing to the use of mantle tissue-specific gene expression profiles the GRN is constrained by the biomineralization process. The treatment of shell-damage and the multifunctional nature of the mantle however, makes it challenging to experimentally differentiate between fundamental biomineral production and other processes in the shell and mantle, namely the immune response. GRN analyses attempt to simplify the interactions of pleiotropic genes and highlight their role in biomineralization; these genes are often obscured in standard differential expression analyses (such as those including environmental stressors) and indeed many of the genes identified in our network analysis were not significant in the differential expression analysis, highlighting the strength of the network approach (Fig. 2, Supplementary Fig. S2). Currently, there are few functional tools available to study any member of the molluscan phyla. Three papers have documented RNAi (Suzuki *et al.*, 2009; Zhao *et al.*, 2016), and genome editing via CRISPR/Cas9 has been reported twice, once in *Crepidula fornicata* (knock-in, Perry and Henry, 2015) and once in *Lymnaea stagnalis* (knock-out, Abe and Kuroda, 2019). Given the lack of tools available to study fundamental questions in this phyla, computational tools are particularly valuable and the data presented here represents a significant resource for the community, which will underpin both hypothesis generation and unbiased discovery methods. The limitations of predicting complex biological interactions from gene expression data alone are well known (Banf and Rhee, 2017), especially in the absence of physical regulatory models such as DNA-binding motifs and chromatin accessibility maps, as is the case here. The GRN resource is an exploratory tool to for unbiased discovery that complements other avenues of investigation. Below we show an example of how the GRN can be used with *in vivo* data to provide insight into regulatory mechanisms, generate new hypotheses and highlight future priority candidates.

### 3.2 Co-expressed genes are clustered by SOTA

SOTA was used to cluster 199 321 Trinity genes into 18 862 expression clusters based on shared expression profiles. We demonstrate that expression clusters generated by SOTA include genes that are co-expressed at the cellular level *in vivo.* For example, expression cluster 2128 includes three of our candidate biomineralization marker genes (*mytilin, tyrA* and *chitin-binding*), which we have previously shown to have overlapping expression in the calcifying epithelium of the mollusc mantle (Fig. 2).

### 3.3 ARACNe predicts interaction between *in vivo* ground truthed biomineralization markers generating unique modules correlated to shell microstructure

Expression cluster profiles from SOTA were used as an input for the network inference algorithm ANACNe, which is an information theory-based approach discriminating between direct and indirect gene-to-gene relationships. Here, we term the resulting relevance network, a computationally predicted GRN. We discovered that our six biomineralization marker genes fall into two modules of the GRN (Fig. 2). The candidates that are all present in the nacreous shell proteome are connected via a mutual first neighbour (unannotated expression cluster 2117, module 1). Likewise, in a separate module, the candidates which are absent from the nacreous proteome, but are still co-expressed at the cellular level in the mantle epithelium, are also connected by an unannotated mutual first neighbour (unannotated expression cluster 2826, module 2). These two unique biomineralization modules suggest that genes involved in the production of the nacreous shell layer are in a separate regulatory network to those of other shell layers, and hence microstructures. Previous immunolocalizations of shell matrix proteins corresponding to specific shell layers and mantle regions have also suggested that different secretory repertoires control the biomineralization processes of each layer (Marie *et al.*, 2012), and in addition, that the mantle organ is modular in nature both at the anatomical and molecular level (Herlitze *et al.*, 2018; Sleight *et al.*, 2016). The computationally predicted GRN data here provide further evidence for both of these hypotheses, in addition to highlighting, for the first time, the possible regulatory networks involved.

### 3.4 Hypothesized functional roles of the biomineralization modules

The two biomineralization GRN modules contained both relatively well-characterized candidate biomineralization genes, in addition to numerous unannotated transcripts. True unbiased genetic screens to test the function of large numbers of genes, for example all of the 1190 genes present in biomineralization modules 1 and 2, are currently impossible in non-model organisms. Instead, annotations were explored using database-based tools, such as StringDB and enrichment analysis. Finally genes were *in silico* screened for priority candidates that we hypothesize are crucial for biomineralization (Table 1).

**Table 1.** Hypothesized functional role and new priority candidates from each of the biomineralization modules in the computationally predicted mantle GRN (Trinity transcript and cluster mapping data available in supplementary informationTable S1).

Module	Putative annotation	Functional group
**Module 1 Functional hypothesis =** *responsible for transcribing and translating the extracellular matrix proteins required for biomineralization of nacreous shell layer (see Supplementary informationTable S2 and Fig.S3)*	Toll-like receptor 2	Receptor and signal transduction
	Toll-like receptor 4	
	Notch 2	
	Headcase protein homolog	
	Forkhead box protein J1	
	FGF receptor 3	
	Thioredoxin domain-containing protein 11	
	Thioredoxin-related transmembrane protein 1	
	Akirin-1	
	Solute carrier family 23 member 1	Ion transport
	Sodium/calcium exchanger 3	
	Pendrin	
	cartilage matrix protein-like	Shell matrix
	Insoluble matrix shell protein 1	
	PIF-like isoform X1	
	PIF-like	
	Carbonic anhydrase 2-like isoform X3	Biomineralization enzyme
	HSP70 12B	Protein folding/chaperoning
	HSP 90A	
	Heat shock factor-binding protein 1	
**Module 2 Functional hypothesis =** *responsible for the transport of both ions and proteins to biomineralization site (see Supplementary informationTable S3 and Fig.S4)*	Notch 2	Receptor and signal transduction
	Toll-like receptor 1	
	Regucalcin	Ion transport
	Solute carrier family 22 member 4	
	Solute carrier family 22 member 5	
	Solute carrier family 23 member 1	
	Solute carrier family 28 member 3	
	Sodium-dependent phosphate transport protein	
	Sodium-coupled monocarboxylate transporter 2	
	Sodium/nucleoside cotransporter 2	
	Sodium/glucose cotransporter 4	
	Alkaline phosphatase, tissue-nonspecific	Biomineralization enzyme
	Tyrosinase	
	Protein transport protein Sec61 alpha-2	Protein transport

The first biomineralization module (module 1) contained four of our biomineralization markers (*pif, mytilin, tryA* and *chitin-binding-domain*) all of which encode proteins that are present in the nacreous shell proteome (Fig. 2). Module 1 comprised of 36 GRN clusters made-up of 1023 trinity genes (Supplementary File S3), 467 of the trinity genes could be assigned putative annotation after sequence similarity searches against NCBI non-redundant, uniref90 and swissprot human databases. Enrichment analysis revealed module 1 was significantly enriched in KEGG pathways, molecular functions (GO), biological processes (GO) and cellular components (GO) related to protein production and turnover (e.g. transcription, translation, ribosomes, Supplementary Fig. S3 and Supplementary Table S2), and we therefore hypothesize this module is responsible for making the extracellular proteins required for repairing and remodelling the damaged shell. Although enrichment analysis suggests the primary function of module 1 is the production of extracellular matrix components, we also found genes in this module related to ion transport. One explanation for the ion transporters in this module could be that they are specific to the nacreous shell layer and creating the correct conditions for nacreous tablet formation, whereas module 2 has a more general role in transport of all ions and proteins for both of the calcified shell layers and the periostracum. From module 1 we have highlighted twenty one priority gene candidates for future characterization (Table 1).

The second biomineralization module (module 2) contained two of our biomineralization markers (*tryB* and *contig01043* *lelliptica mantle edge gene’*) which have specific expression in the mantle epithelial/shell secreting cells but are absent from the nacreous shell proteome (Fig. 2). Module 2 comprised of 23 GRN clusters made-up of 167 trinity genes (Supplementary File S4), 63 of the trinity genes could be assigned putative annotation after sequence similarity searches. Further exploration of these annotations revealed the module was functionally enriched in ion and protein transport genes, as well as containing enzymes that catalyze mineralization and possible upstream receptors and ion exchange regulators (Supplementary Fig. S4 and Supplementary Table S3). Taken together with the lack of presence in the nacreous shell proteome, we hypothesize that the primary functional role of module 2 is to orchestrate the transport of both ions and proteins to the shell secretion site, which are required during normal shell growth, and repair. From module 2 we have highlighted fourteen biomineralization priority gene candidates for future characterization (Table 1).

### 3.5 *In silico* screen for priority candidates for future genome editing in the mollusca

Both of the biomineralization modules were *in silico* screened for five functional categories of genes: receptors and signal transduction, ion transport, shell matrix proteins, biomineralization enzymes and protein folding/chaperoning, in addition to literature searches for genes known to be involved in biomineralization in other systems. Using this two-step screen criteria we were able to identify genes novel to the context of biomineralization (such as *toll-like receptors*), as well as genes that have a well-established role in biomineralization in other organisms but, have not previously reported in molluscs (such as *SLC23A1*).

#### 3.5.1 Receptors and signal transduction

The presence of receptors in molluscan shell proteomes was recently discussed by Herlitze *et al.* (2018), it is speculated that signalling molecules could be incorporated into the shell to allow the acellular structure a means by which to communicate with the underlying mantle epithelium. If the shell is damaged, for example, these signalling molecules would be released and detected by the mantle, triggering the upregulation of calcification to repair the shell. In addition to being a possible means for feedback between the extracellular shell and the mantle, receptors are likely to be pivotal in the regulation of mantle modularity, normal shell growth, and repair. Genes encoding receptors and proteins involved in signal transduction were in both biomineralization modules. Four of these were *toll-like receptors* that have been previously found in many molluscan mantle transcriptomes, where they are hypothesized to be functioning as pattern recognition receptors for the identification of pathogens as part of the innate immune system (Zheng *et al.*, 2005). More recently toll-like receptors have also been identified in shell proteomes, where they have been speculated to be a part of a phenoloxidase precursor to immunity pathways such as melanization, phagocytosis, capsulation, opsonization (Arivalagan *et al.*, 2017).

Receptors and signal transduction genes important in development and regeneration were also identified in both of the biomineralization modules. Two candidates were putatively annotated as *notch2*, a cell-cell communication receptor widely important for metazoan (including molluscan) development (De Oliveira *et al.*, 2016), and more specifically pivotal in bone development and regeneration in vertebrates (Shao *et al.*, 2018). *Notch2* has also been reported in the developing shell gland of a gastropod mollusc, *C.fornicata* (Perry *et al.*, 2015). Further inspection of the *notch2* alignments here however, revealed the transcripts contained a repeated string of epidermal growth factor (EGF) motifs matching just the extracellular domain of notch, rather than the full complement domains. We therefore speculate the new candidates could be novel receptors involved in cell-cell communication. Two additional genes that are known to be involved in the development of vertebrate teeth and bones were highlighted from module 1; *forkhead box protein J1* (Jheon *et al.*, 2013) and *fibroblast growth factor receptor 3* (Colvin *et al.*, 1996; Tapaltsyan *et al.*, 2016). The expression of other *fox* genes, such as *foxa*, have been documented in the gastropod developing shell gland (Perry *et al.*, 2015), but to our knowledge there are very little data to date on the involvement of classic vertebrate biomineral development genes in the regulation of biomineralization in molluscs. Given a number of these vertebrate genes were found in both of the biomineralization modules presented here, we speculate they could be involved in the development and regeneration of molluscan shell, highlighting the requirement for more functional testing and comparative work to elucidate a possible deeply conserved metazoan biomineralization GRN.

#### 3.5.2 Ion transport

In order to calcify the shell, molluscs must transport calcium ions across the mantle tissue to the extrapallial fluid and shell depositions site (Sillanpää *et al.*, 2018). Genes that are likely to be involved in this transport process were found in both of the biomineralization modules. Three ion transport candidates found in module 1, all of which transport ions potentially relevant for molluscan biomineralision. Firstly, *solute carrier family 23 member 1* (*SLC23A1*) is a vitamin C transporter which mediates the uptake of Vitamin C in exchange for sodium ions. In vertebrate bone Vitamin C is an essential cofactor for collagen assembly (Aghajanian *et al.*, 2015), and in addition, recent studies in coral larvae show *SLC23A1* it is the single most up-regulated gene at the onset of larval calcification (Rosenthal *et al.*, 2016). Secondly, *sodium/calcium exchanger 3* is an antiporter membrane protein that removes calcium from cells, it has been proposed as a candidate for biomineralization in both echinoderms (Flores and Livingston, 2017) and molluscs (Shi *et al.*, 2013). And thirdly, *pendrin* (*SLC26A4*) is a sodium-independent transporter of various ions, including bicarbonates required for shell biomineralization. Mutations to the pendrin gene in mice and humans lead to a variety of deafness phenotypes, such as reduced calcification of the inner ear bones (Dror *et al.*, 2014).

Module 2 contained eight biomineralization candidates putatively annotated as ion transporters. Similar to module 1, some of these ion transporters have previously been related to biomineralization in other systems, whereas some are novel in the context on biomineralization. In addition to the ion channel/transport genes module 2 also contained regucalcin. Regucalcin is pivotal to the regulation of calcium ion homeostasis and biomineralization in vertebrates (Yamaguchi, 2014) and it is highly expressed in other mollusc mantle transcriptomes (Li *et al.*, 2017). We speculate that similar to vertebrates, regucalcin could be acting as a regulator of ion transport activity in this module.

#### 3.5.3 Protein transport

Similar to the transport of ions, proteins in the extracellular matrix of the shell also need to be transported across the mantle to the shell deposition site, and therefore we screened the two biomineralization modules for protein transport genes. Module 2 included one gene putatively annotated as a protein transporter, *sec61 alpha-2*, which is a component of the sec dependent transport pathway. This pathway is a general, highly conserved, protein export system, transporting newly synthesized proteins into or across the cell membrane (Linxweiler *et al.*, 2017; Rapoport, 2007). We hypothesize molluscs could use this pathway to transport extracellular matrix proteins across the mantle epithelial membrane, to the extrapallial fluid and biomineralization site.

#### 3.5.4 Protein folding/chaperoning

The role of protein folding and heat shock proteins (HSPs) in molluscan biomineralization is relatively unexplored. Our previous work has demonstrated high levels of *hsp* gene expression, both constitutively and induced in response to shell damage and heat stress, in the mantle tissue of two bivalve species (*L. elliptica* and *Mya truncata*) (Clark *et al.*, 2010; Sleight *et al.*, 2015, 2016, 2018). In the present study, the computationally predicted GRN allocated three hsps into module 1: *Heat shock 70 kDa protein 12B*, *HSP 90-alpha*, *Heat shock factor-binding protein 1*, leading us to hypothesize that hsps are important in the folding and chaperoning of shell matrix proteins during shell repair and remodelling. In addition to our own work on marine invertebrates, it was recently demonstrated in mice that *HSP 90-alpha* is secreted and directly influences remodelling of the extracellular matrix during wound repair (Bhatia *et al.*, 2018).

#### 3.5.5 Biomineralization-specific enzymes

Enzymes are crucial for the production of all biominerals (Weiss and Marin, 2008), they can be categorized in many different ways based on type of biochemical catalytic activity, or biological function carried out, from protein synthesis to signalling cascades, ion transport and mineral nucleation. Here we describe the genes in modules 1 and 2 encoding enzymes that can be categorized as specific to mineralization [according to Weiss and Marin (2008)].

Carbonic anhydrase catalyzes the hydration of carbon dioxide and accelerates the formation of bicarbonate ions, it is thought of as a deeply conserved requirement for biomineralization across many taxa (Le Roy *et al.*, 2014) and carbonic anhydrase domains are often components of multi-functional structural matrix proteins, for example nacrein (Miyamoto *et al.*, 1996). In module 1 we found one gene encoding a carbonic anhydrase, we hypothesize this enzyme is therefore synthesized and secreted, along with other shell matrix proteins in this module, into the extrapallial space, where it catalyzes the production of bicarbonate ions required for mollusc shell mineralization.

Similar to carbonic anhydrase, alkaline phosphatase is common to many mineralizing taxa, it hydrolyses pyrophosphate and provides inorganic phosphate ions to promote mineralization (Golub and Boesze-Battaglia, 2007). Alkaline phosphatase activity plays a role in molluscan shell production, and its activity can be used a marker of biomineralizing cells (Hohagen and Jackson, 2013).We found *alkaline phosphatase* in module 2, along with other regulators of ions such as, ion transporters, and therefore highlight it as an important component of biomineralization governed by the regulatory genes in module 2.

Contrary to the two enzymes discussed above that catalyze ion production, tyrosinase enzymes catalyze the production of quinones. Quinones then bind amino acids to form protein cross-linkages leading to the formation of insoluble proteins, such as those present in the uncalcified proteinaceous cuticle layer covering the molluscan shell, the periostracum (Aguilera *et al.*, 2014). The insoluble periostracum seals the extrapallial space to isolate it from the external environment, enabling super saturation conditions inside. It has also been speculated as an initial substrate for biomineralization, in addition to being critically important for shell damage-repair as a means to seal the damaged area before remodelling and new mineralization can occur. Tyrosinase domains have been proposed as evolutionary maintained and conserved components required for all molluscan biomineralization (Arivalagan *et al.*, 2017), and we have previously characterized two *tyrosinases* in the *L. elliptica* mantle transcriptome, which show similar spatial expression patterns in the mantle epithelium, but only one, *tyrA* has a translated protein product present in the shell proteome (Fig. 2). The two previously characterized *tryrosinase* genes were used as biomineralization markers in this GRN and we discovered that *tryA* was included in module 1, along with other shell matrix proteins in the nacreous shell proteome, whereas *tryB* was included in module 2 along with other biomineralization markers absent from the proteome. The inclusion of the two copies into different biomineralization modules further supports the hypothesis that this gene duplication was followed by sub-functionalization. The presence of an additional transcript with sequence similarity to tyrosinase in module 2 indicates there are more than two copies in *L. elliptica*, which is perhaps not surprising as many mollusc species have large gene expansions of this family (Aguilera *et al.*, 2014) and we sequenced 78 individual mantle tissues significantly increasing the RNA-Seq data for this species, hence increasing our likelihood of finding novel genes.

#### 3.5.6 Shell matrix

In addition to the four previously characterized nacreous shell matrix proteins present in module 1, we highlight four additional transcripts which are also likely to encode shell matrix proteins, but were not previously found in *L. elliptica* shell proteome or mantle transcriptome studies.

#### 3.5.7 Unannotated genes

The two biomineralization modules contained a total of 660 transcripts that had no sequence similarity to anything in the four databases searched (556 transcripts in module 1 and 104 transcripts in module 2). Given we cannot use database methods to assess the likely functional role of these genes it is difficult to highlight specific candidates of interest for future work. Clearly these unannotated genes are important for biomineralization, as our network analysis has included them in biomineralization modules of the GRN and we therefore propose another screening criteria for unannotated genes so that they can also be included in future functional work. Firstly the time-dependent damage-response analysis could be used to filter the list of unannotated genes, and secondly genes with a high threshold of connectivity to our biomineralization markers and new priority candidates could be selected.

## Supplementary Material

btz754_Supplementary_Data
