# Supplementary material for: Computationally predicted gene regulatory networks in molluscan biomineralization identify extracellular matrix production and ion transportation pathways
Source: Bioinformatics. 2019 Oct 16;36(5):1326–32. doi: 10.1093/bioinformatics/btz754 (PMC7703775; doi:10.1093/bioinformatics/btz754)
Supplement: btz754_Supplementary_Data [file btz754_supplementary_data.zip › btz754-Suppl_Data/SLEIGHT_etal_Supplementary_information_Figs&tables_REVISED_R3_for_final_article.pdf]

Supplementary information

Systems Biology

Computationally Predicted Gene Regulatory Networks in Molluscan Biomineralization Identify Extracellular Matrix Production and Ion Transportation Pathways

Victoria A. Sleight[1,2,*], Philipp Antczak[3], Francesco Falciani[3] and Melody S. Clark [2]

[1]Department of Zoology, Address University of Cambridge, Downing Street, Cambridge [2]British Antarctic Survey, Address High Cross, Madingley Road, Cambridge, [3]Department of Functional and Comparative Genomics, Address Institute of Integrative Biology, University of Liverpool, Biosciences Building, Liverpool.

*To whom correspondence should be addressed.

Abstract

Motivation: The molecular processes regulating molluscan shell production remain relatively uncharacterised, despite the clear evolutionary and societal importance of biomineralization.

Results: Here we built the first computationally predicted gene regulatory network (GRN) for molluscan biomineralization using Antarctic clam (*Laternula elliptica*) mantle gene expression data produced over an age-categorised shell damage-repair time-course. We used previously published *in vivo in situ* hybridisation expression data to ground truth gene interactions predicted by the GRN and show that candidate biomineralization genes from different shell layers, and hence microstructures, were connected in unique modules. We characterised two biomineralization modules of the GRN and hypothesise that one module is responsible for translating the extracellular proteins required for growing, repairing or remodelling the nacreous shell layer, whereas the second module orchestrates the transport of both ions and proteins to the shell secretion site, which are required during normal shell growth, and repair. Our findings demonstrate that unbiased computational methods are particularly valuable for studying fundamental biological processes and gene interactions in non-model species where rich sources of gene expression data exist, but annotation rates are poor and the ability to carry out true functional tests are still lacking.

Contact: vas45@cam.ac.uk

Supplementary information: Supplementary data are available at *Bioinformatics* online.

Supplementary Methods

RNA-Seq and gene abundance estimation

Stranded cDNA libraries (n = 78) were prepared using the NEXTflex™ Rapid Illumina Directional RNA-Seq Library Prep Kit and sequencing was carried out over 5 lanes on a Hi-Seq 2000 generating 125 base paired-end reads.

All libraries were used to *de novo* assemble a representative mantle transcriptome (available in Supplementary File 1). Briefly, adaptor sequences and ribosomal RNA reads were removed, and resulting reads were further cleaned for quality (Phred score 30) and minimum read length (80 bp) using the ea-utils tool (v1.1.2) fastq-mcf. The cleaned reads were normalised using Trinity's (v2.2.0) *in silico* Read Normalisation tool (Haas, *et al.*, 2013), with default parameters. Normalised reads were *de novo* assembled using Trinity (v2.2.0) with default parameters (Grabherr, *et al.*, 2011). The longest isoform of each gene was extracted from the transcriptome for annotation as per An *et al.* (2014) using the Trinity utility script get_longest_isoform_seq_per (assembly statistics and assembled transcripts available for download in Supplementary File 1). The longest isoforms of each gene were compared to a local NCBI non-redundant (nr) database (updated 01 June 2016) using Basic Local Alignment Search Tool (blastx, cut-off $<1e^{-10}$) to search for sequence similarity and putative gene annotation (Altschul, *et al.*, 1990).

Transcript abundance was estimated by alignment-based quantification using Trinity (v2.2.0) utilities (Grabherr, *et al.*, 2011; Haas, *et al.*, 2013). Transcripts were aligned to the *de novo* transcriptome using bowtie with default parameters and transcript abundance estimates were calculated using RNA-Seq by Expectation-Maximization (RSEM). A matrix of Trimmed Mean of M-values [TMM] normalised Fragments Per Kilobase Of Exon Per Million Fragments Mapped [FPKM] values was loaded into TM4 MultiExperiment Viewer (Howe, *et al.*, 2011) and clustered based on expression profiles for each gene using SOTA (Herrero, *et al.*, 2001).

ARACNe prediction of GRN

To construct a predicted GRN from the SOTA expression cluster profiles, ARACNe was implemented using "ARACNe for GNU/Linux" with a p-value cut-off of $1e^{-7}$ and DPI set to 0.1 (Margolin, *et al.*, 2006). The ARACNe network output (Supplementary file 2) was loaded in Cytoscape v3.4.0 for visualisation and exploration (Shannon, *et al.*, 2003). To identify highly interconnected sub-networks, GLay (clusterMaker) was applied (Supplementary information Fig.S1, (Morris, *et al.*, 2011)). Gene annotations (Blastx nr, as above) were mapped onto nodes in the regulatory gene network.

In vivo ground truth of biomineralization modules

We have previously identified and characterised biomineralization gene candidates in the Antarctic clam using a combination of semi-qPCR, *in situ* hybridisation (ISH) and proteomics (Sleight, *et al.*, 2016). Six of these candidate genes whose expression is specific to the calcifying outer epithelium of the mollusc mantle can be split into two groups. One set of translated proteins are also present in the nacreous shell proteome (*tyrA, chitin-binding domain, mytilin, pif*), whilst a second set do not have a translated protein detected in the nacreous shell proteome (*tyrB* and *Contig01043-unannotated transcript,* summarised in Fig. 2). These six candidate biomineralization genes were used as molecular markers of biomineralization, they

were firstly all confirmed to be present in the current data set, and were then investigated in the ARACNe predicted GRN to test for predictions of co-expression and interaction via first and second neighbour connections.

Differential expression analysis

The gene-level abundance estimates (raw counts) for each of the libraries were constructed into a matrix for downstream expression analyses (using the Trinity abundance_estimates_to_matrix.pl script). Differentially expressed genes were identified using the Bioconductor (v3.4) edgeR package in R (v3.1.1) with a false discovery rate (FDR) of 5% (Robinson *et al.* 2010, McCarthy *et al.* 2012) and genes which showed significant temporal changes in response to damage, termed here, time-dependant damage-response genes, were identified. Genes that qualified as a time-dependent damage response gene in any of the three age categories were mapped onto the network. To investigate if the biomineralization modules and priority candidates were enriched in time-dependent damage-response genes, all genes were ranked by FDR and the distributions of significant genes were assessed for different module conditions in each age category.

Enrichment analysis and identification of priority candidates using biomineralization markers

The two modules that contained our previously defined biomineralization markers were investigated. All of the trinity transcripts within each module were extracted and re-annotated using three databases: NCBI nr, Uniref90 and Swissprot human (Supplementary Files 3 & 4). Annotations were then analysed using StringDB and tested for enrichment against a whole genome background. In order to perform an *in silico* screen for priority candidate genes within the two biomineralization modules two criteria were used: firstly, genes which had GO terms relating to functional categories with potential relevance to biomineralization were identified (categories = receptors and signal transduction, ion transport, shell matrix proteins, biomineralization enzymes and protein folding/chaperoning) and secondly, using literature searches, each gene was assessed to identify if it had previously been reported to play a role in the regulation of biomineralization in other systems.

Supplementary Results: Figures and Tables

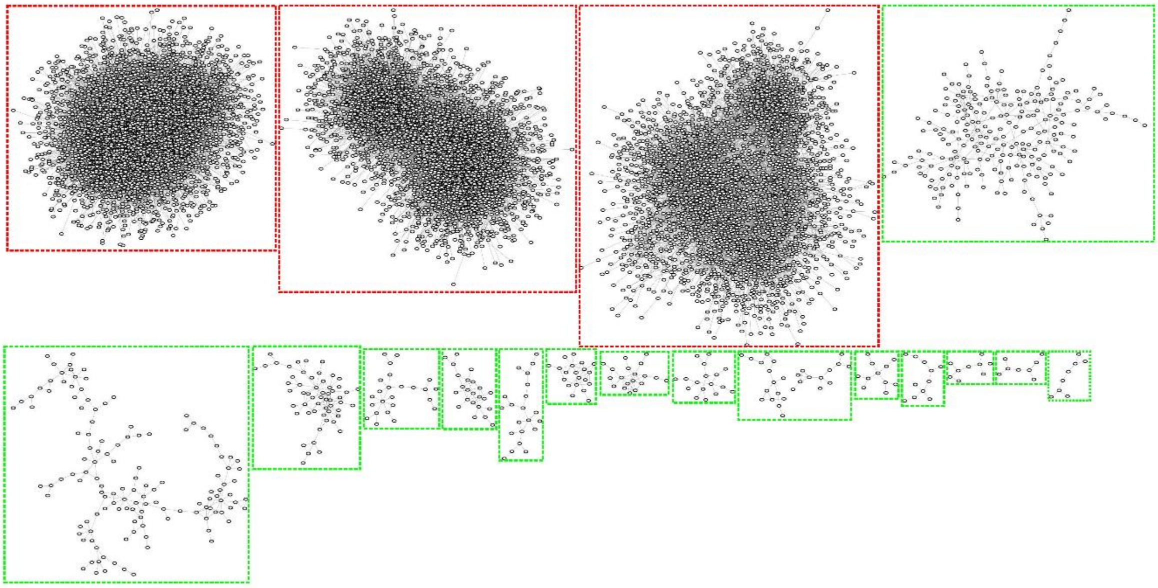

Fig.S1. *Laternula elliptica* mantle tissue gene regulatory network created using ARACNE (p-value cut-off 1e^{-7}) consisting of 13,577 nodes. Highly interconnected regions identified using GLay yielding three large and fifteen small expression clusters, "large" clusters highlighted in red and are also represented in Fig. 2 of main manuscript and "small" highlighted in green. The network file is available for download in Supplementary File 2.

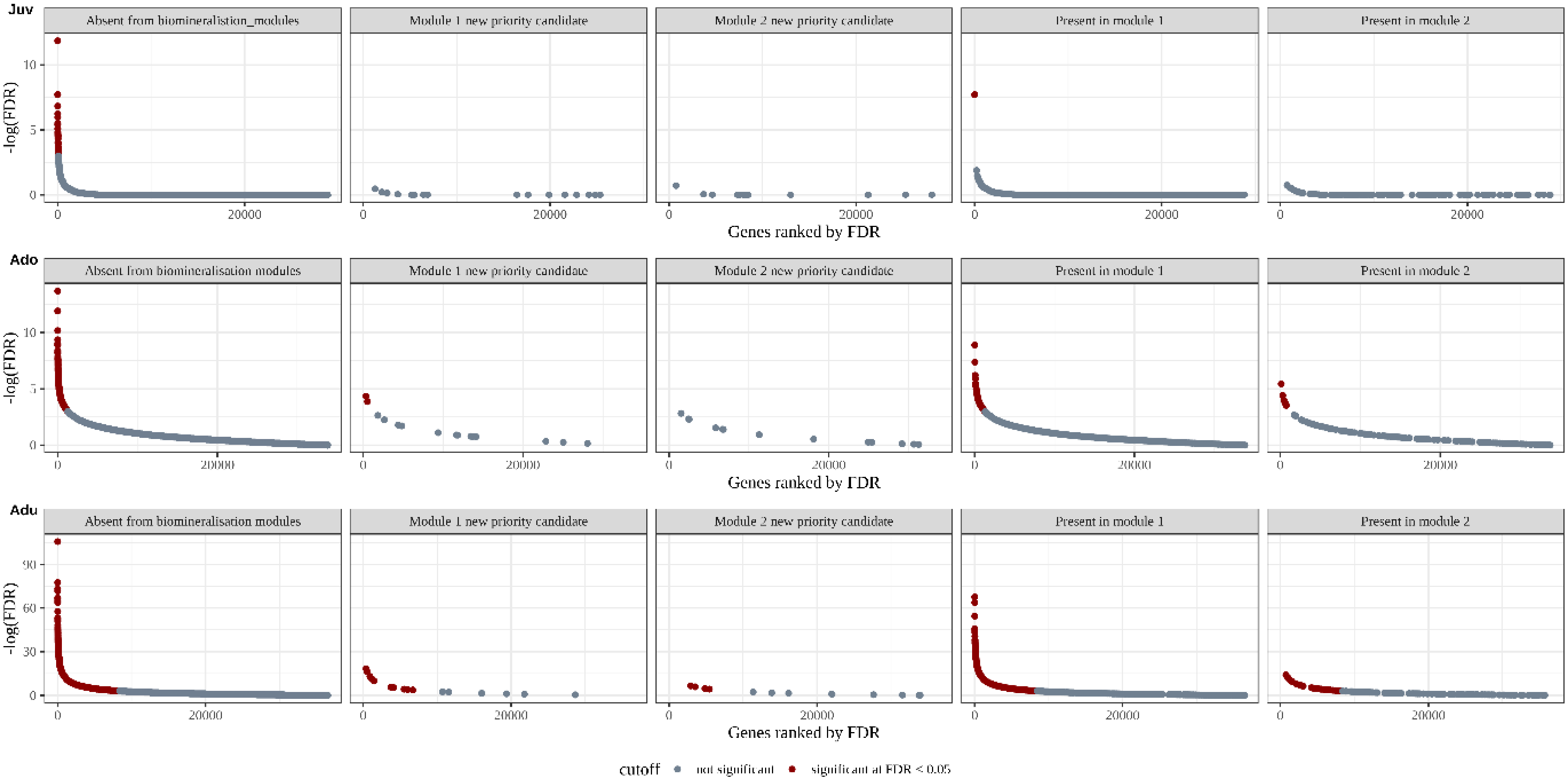

Fig.S2. Assessment of time-dependent damage-response genes in each age category in the GRN. Biomineralization modules of the GRN and the priority gene lists do not contain a higher proportion of significant time-dependent damage-response genes (red).

Table S1. Hypothesised functional role and new priority annotated candidates from each of the biomineralization modules in the computationally predicted mantle GRN, including Trinity ID and cluster mapping information, colour corresponds to functional group mapping of Fig. 2 in main manuscript.

Module	GRN Cluster ID	Trinity ID	Putative annotation	Functional group
**Module 1 Functional hypothesis =** *responsible for transcribing and translating the extracellular rmatrix proteins required for biomineralization of nacreous shell layer (see Supplementary information Table S1 and Fig.S3)*	Cluster-9690.txt	TRINITY_DN255896_c3_g2	Toll-like receptor 4	Receptor and signal transduction
	Cluster-9690.txt	TRINITY_DN245288_c2_g1	Toll-like receptor 2	
	Cluster-2849.txt	TRINITY_DN246350_c2_g1	Toll-like receptor 4	
	Cluster-2128.txt	TRINITY_DN248655_c2_g5	Neurogenic locus notch homolog protein 2	
	Cluster-10028.txt	**TRINITY_DN251622_c0_g1**	Headcase protein homolog	
	Cluster-10469.txt	TRINITY_DN251675_c0_g1	Forkhead box protein J1	
	Cluster-2128.txt	TRINITY_DN253939_c2_g1	Fibroblast growth factor receptor 3	
	Cluster-18226.txt	TRINITY_DN242791_c2_g1	Thioredoxin domain-containing protein 11	
	Cluster-9587.txt	**TRINITY_DN256751_c1_g1**	Thioredoxin-related transmembrane protein 1	
	Cluster-15239.txt	TRINITY_DN246576_c0_g1	Akirin-1	
	Cluster-2346.txt	**TRINITY_DN259824_c2_g1**	Solute carrier family 23 member 1	Ion transport
	Cluster-10050.txt	**TRINITY_DN256986_c3_g4**	Sodium/calcium exchanger 3	
	Cluster-10335.txt	TRINITY_DN246390_c1_g1	Pendrin	
	Cluster-13458.txt	**TRINITY_DN251420_c3_g1**	cartilage matrix protein-like	Shell matrix
	Cluster-12543.txt	**TRINITY_DN242528_c0_g1**	Insoluble matrix shell protein 1	
	Cluster-9662.txt	**TRINITY_DN250104_c5_g8**	PIF-like isoform X1	
	Cluster-9665.txt	**TRINITY_DN244470_c1_g2**	PIF-like	
	Cluster-2079.txt	TRINITY_DN256420_c0_g2	Carbonic anhydrase 2-like isoform X3	Biomineralization enzyme
	Cluster-10478.txt	TRINITY_DN239234_c0_g1	Heat shock 70 kDa protein 12B	Protein folding/chaperoning
	Cluster-9701.txt	**TRINITY_DN249833_c1_g2**	Heat shock protein HSP 90-alpha	
	Cluster-16489.txt	**TRINITY_DN255921_c0_g1**	Heat shock factor-binding protein 1	
**Module 2 Functional hypothesis =** *responsible for the transport of both ions and proteins to biomineralization site (see Supplementary information Table S2 and Fig.S4)*	Cluster-3198.txt	TRINITY_DN259845_c3_g3	Alkaline phosphatase, tissue-nonspecific	Biomineralization enzyme
	Cluster-2772.txt	TRINITY_DN256081_c2_g5	Tyrosinase	
	Cluster-71.txt	**TRINITY_DN253708_c3_g3**	Neurogenic locus notch homolog protein 2	Receptor
	Cluster-2764.txt	**TRINITY_DN248447_c0_g2**	Toll-like receptor 1	
	Cluster-6852.txt	TRINITY_DN255895_c0_g3	Protein transport protein Sec61 alpha-2	Protein transport
	Cluster-687.txt	TRINITY_DN244879_c4_g1	Regucalcin	Ion transport
	Cluster-6827.txt	TRINITY_DN237157_c0_g1	Sodium/glucose cotransporter 4	
	Cluster-2631.txt	**TRINITY_DN231473_c0_g2**	Sodium/nucleoside cotransporter 2	
	Cluster-2568.txt	TRINITY_DN239292_c0_g1	Sodium-coupled monocarboxylate transporter 2	
	Cluster-606.txt	TRINITY_DN247229_c2_g1	Sodium-dependent phosphate transport protein	
	Cluster-596.txt	TRINITY_DN253103_c2_g3	Solute carrier family 22 member 4	
	Cluster-10275.txt	**TRINITY_DN255933_c0_g1**	Solute carrier family 22 member 5	
	Cluster-2690.txt	**TRINITY_DN245321_c4_g2**	Solute carrier family 23 member 1	
	Cluster-2652.txt	TRINITY_DN250916_c0_g3	Solute carrier family 28 member 3	

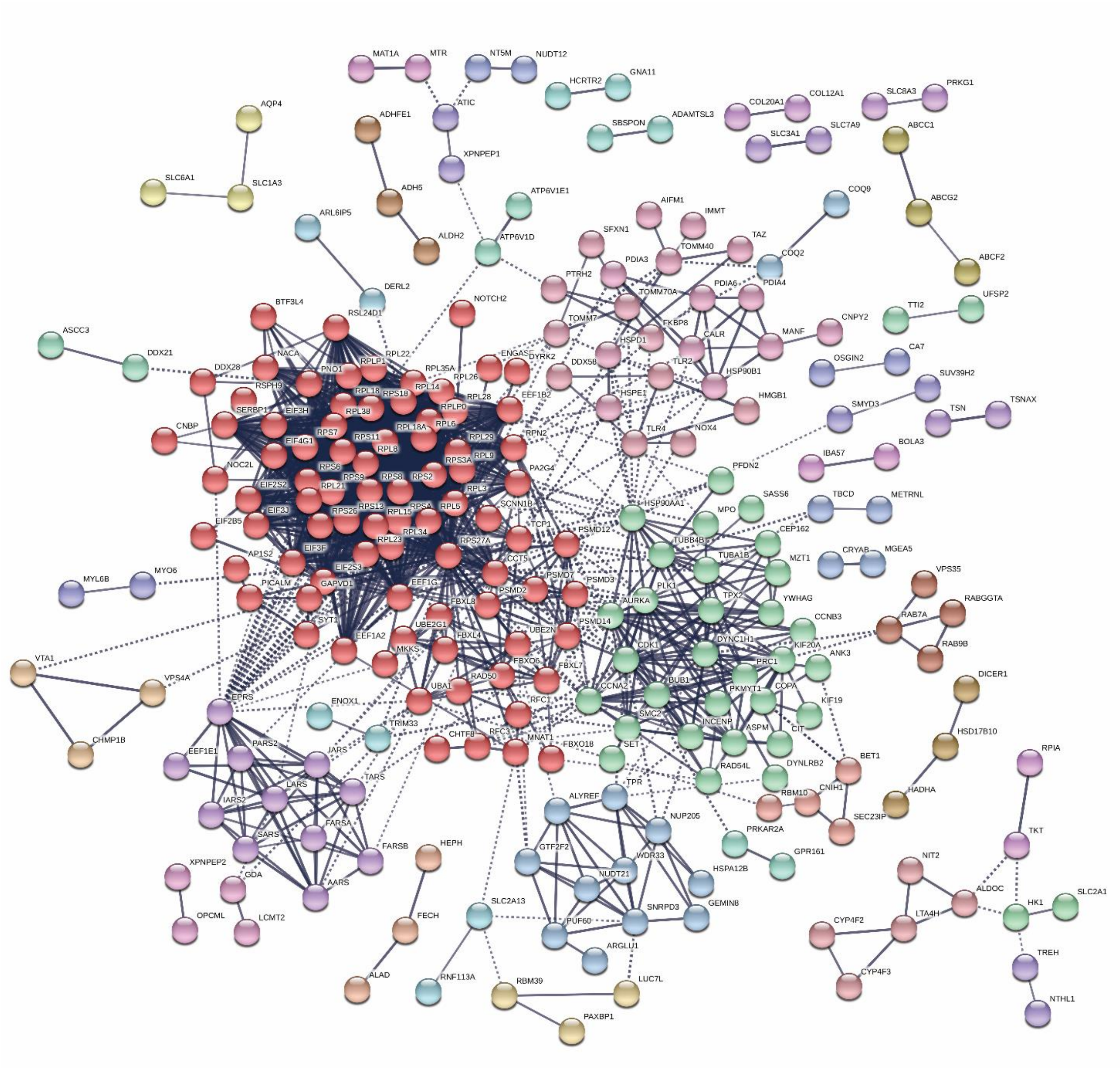

Fig.S3. StringDB predicted protein interactions for all swissprot (human) annotated Trinity genes in GRN module 1 (Cluster-2730 1st and 2nd neighbours). Node colours represent clustering (Markov Cluster Algorithm [MCL], inflation parameter = 2), weight of line indicates confidence of interaction, more detailed information on annotations can be found in corresponding supplementary file.

Table S1. Functional enrichment analysis, against whole genome background, of String DB network generated from module 1 (Cluster-2730 1st and 2nd neighbours).

KEGG			
pathway ID	pathway description	observed gene count	false discovery rate
3010	Ribosome	38	2.36E-31
970	Aminoacyl-tRNA biosynthesis	10	1.6E-06
3013	RNA transport	12	0.00588
4141	Protein processing in endoplasmic reticulum	12	0.00866
4145	Phagosome	11	0.00891
3050	Proteasome	6	0.0104
Molecular Function (GO) – most significant 10 only			
pathway ID	pathway description	observed gene count	false discovery rate
GO.0003735	structural constituent of ribosome	32	9.37E-23
GO.0003723	RNA binding	91	5.78E-19
GO.0044822	poly(A) RNA binding	74	1.51E-16
GO.0003824	catalytic activity	171	1.03E-12
GO.0005198	structural molecule activity	43	2.92E-11
GO.1901363	heterocyclic compound binding	167	3.13E-09
GO.0097159	organic cyclic compound binding	167	8.30E-09
GO.0036094	small molecule binding	98	1.94E-08
GO.0000166	nucleotide binding	88	2.09E-07
GO.0004812	aminoacyl-tRNA ligase activity	10	2.14E-06
Biological Process (GO) – most significant 10 only			
pathway ID	pathway description	observed gene count	false discovery rate
GO.0006613	cotranslational protein targeting to membrane	39	2.74E-34
GO.0045047	protein targeting to ER	39	2.74E-34
GO.0019083	viral transcription	39	4.16E-34
GO.0006614	SRP-dependent cotranslational protein targeting to membrane	38	8.28E-34
GO.0070972	protein localization to endoplasmic reticulum	41	8.28E-34
GO.0019080	viral gene expression	39	5.84E-33
GO.0000184	nuclear-transcribed mRNA catabolic process, nonsense-mediated decay	38	2.04E-32
GO.0006612	protein targeting to membrane	41	6.41E-30
GO.0006413	translational initiation	44	9.12E-28
GO.0006412	translation	50	1.79E-26
Cellular Component (GO) – most significant 10 only			
pathway ID	pathway description	observed gene count	false discovery rate
GO.0022626	cytosolic ribosome	34	1.18E-31
GO.0044445	cytosolic part	37	5.94E-25
GO.0044391	ribosomal subunit	33	1.57E-24
GO.0044444	cytoplasmic part	237	2.68E-22
GO.0005829	cytosol	140	1.89E-20
GO.0005840	ribosome	34	1.96E-19
GO.0070062	extracellular exosome	122	4.75E-17
GO.0022625	cytosolic large ribosomal subunit	18	9.31E-17
GO.0022627	cytosolic small ribosomal subunit	16	1.33E-16
GO.0005737	cytoplasm	272	4.09E-16

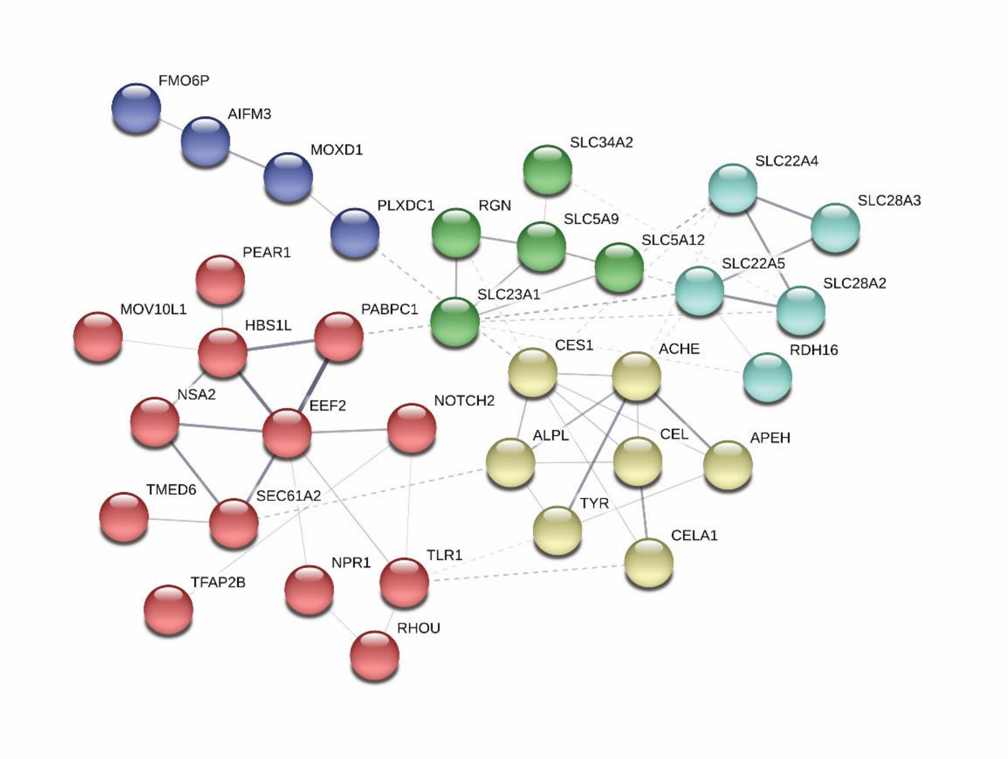

Fig.S4. StringDB predicted protein interactions for all swissprot (human) annotated Trinity genes in GRN module 2 (Cluster-2775 1st and 2nd neighbours). Node colours represent clustering (Markov Cluster Algorithm [MCL], inflation parameter = 2), weight of line indicates confidence of interaction, more detailed information on annotations can be found in corresponding supplementary file.

Table S3. Functional enrichment analysis, against whole genome background, of String DB network generated from module 2 annotations

	Biological Process (GO)		
pathway ID	pathway description	observed gene count	false discovery rate
GO.0006814	sodium ion transport	8	0.0000195
	Molecular Function (GO)		
pathway ID	pathway description	observed gene count	false discovery rate
GO.0015293	symporter activity	7	0.0000549
GO.0005415	nucleoside:sodium symporter activity	2	0.00845
GO.0015226	carnitine transmembrane transporter activity	2	0.00845
GO.0008028	monocarboxylic acid transmembrane transporter activity	3	0.0205
GO.0015077	monovalent inorganic cation transmembrane transporter activity	6	0.0205
GO.0015205	nucleobase transmembrane transporter activity	2	0.0205
GO.0015294	solute:cation symporter activity	4	0.0205
GO.0046943	carboxylic acid transmembrane transporter activity	4	0.0205
GO.1901618	organic hydroxy compound transmembrane transporter activity	3	0.0205
GO.0008509	anion transmembrane transporter activity	5	0.0217
GO.0052689	carboxylic ester hydrolase activity	4	0.0217
GO.0015370	solute:sodium symporter activity	3	0.026
GO.0015211	purine nucleoside transmembrane transporter activity	2	0.0261
GO.0015081	sodium ion transmembrane transporter activity	4	0.0286
GO.0016787	hydrolase activity	13	0.0443

<u>References</u>

Altschul,S.F. *et al.* (1990) Basic local alignment search tool. *J. Mol. Biol.*, **215**(3), 403-410.

An,J. *et al.* (2014) Transcriptome profiling to discover putative genes associated with paraquat resistance in Goosegrass (*Eleusine indica* L.). *Plos One*, **9**(6), e99940.

Grabherr,M.G. *et al.* (2011) Full-length transcriptome assembly from RNA-Seq data without a reference genome. *Nature Biotechnology*, **29**(7), 644-U130.

Haas,B.J. et al. (2013) De novo transcript sequence reconstruction from RNA-Seq: reference generation and analysis with Trinity. Nat. Protoc., **8**(30), 1494

Howe,E.A. *et al.* (2011) RNA-Seq analysis in MeV. *Bioinformatics*, **27**(22), 3209-3210.

McCarthy,D.J. *et al.* (2012) Differential expression analysis of multifactor RNA-Seq experiments with respect to biological variation. *Nucleic Acids Res.*, **40**(10), 4288-4297.

Morris,J.H. *et al.* (2011) clusterMaker: a multi-algorithm clustering plugin for Cytoscape. *BMC Bioinformatics*, **12**, 436.

Robinson,M.D. *et al.* (2010) edgeR: a Bioconductor package for differential expression analysis of digital gene expression data. *Bioinformatics*, **26**(1), 139-140.

Shannon,P. *et al.* (2003) Cytoscape: A software environment for integrated models of biomolecular interaction networks. *Genome Research*, **13**(11), 2498-2504.

Sleight,V.A. *et al.* (2016) An Antarctic molluscan biomineralisation tool-kit. *Sci. Rep.*, **6**, 36978.
