# Supplementary material for: Computationally predicted gene regulatory networks in molluscan biomineralization identify extracellular matrix production and ion transportation pathways
Source: Bioinformatics. 2019 Oct 16;36(5):1326–32. doi: 10.1093/bioinformatics/btz754 (PMC7703775; doi:10.1093/bioinformatics/btz754)
Supplement: btz754_Supplementary_Data [file btz754_supplementary_data.zip › btz754-Suppl_Data/SLEIGHT_etal_Supplementary_information_Figs&tables_REVISED_R2.docx]

| Systems Biology  Computationally Predicted Gene Regulatory Networks in Molluscan Biomineralisation Identify Extracellular Matrix Production and Ion Transportation Pathways  Victoria A. Sleight^1,2,*^, Philipp Antczak^3^, Francesco Falciani^3^ and Melody S. Clark ^2^  ^1^Department of Zoology, Address University of Cambridge, Downing Street, Cambridge ^2^British Antarctic Survey, Address High Cross, Madingley Road, Cambridge, ^3^Department of Functional and Comparative Genomics, Address Institute of Integrative Biology, University of Liverpool, Biosciences Building, Liverpool.  *To whom correspondence should be addressed.  Abstract  **Motivation:** The molecular processes regulating molluscan shell production remain relatively uncharacterised, despite the clear evolutionary and societal importance of biomineralisation.  **Results:** Here we built the first computationally predicted gene regulatory network (GRN) for molluscan biomineralisation using Antarctic clam (*Laternula elliptica*) mantle gene expression data produced over an age-categorised shell damage-repair time-course. We used previously published *in vivo in situ* hybridisation expression data to ground truth gene interactions predicted by the GRN and show that candidate biomineralisation genes from different shell layers, and hence microstructures, were connected in unique modules. We characterised two biomineralisation modules of the GRN and hypothesise that one module is responsible for translating the extracellular proteins required for growing, repairing or remodelling the nacreous shell layer, whereas the second module orchestrates the transport of both ions and proteins to the shell secretion site, which are required during normal shell growth, and repair. Our findings demonstrate that unbiased computational methods are particularly valuable for studying fundamental biological processes and gene interactions in non-model species where rich sources of gene expression data exist, but annotation rates are poor and the ability to carry out true functional tests are still lacking.  **Contact:** vas45@cam.ac.uk  **Supplementary information:** Supplementary data are available at *Bioinformatics* online. |
| --- |

**Supplementary information**

**Supplementary Methods**

RNA-Seq and gene abundance estimation

Stranded cDNA libraries (n = 78) were prepared using the NEXTflex™ Rapid Illumina Directional RNA-Seq Library Prep Kit and sequencing was carried out over 5 lanes on a Hi-Seq 2000 generating 125 base paired-end reads.

All libraries were used to *de novo* assemble a representative mantle transcriptome (available in Supplementary File 1). Briefly, adaptor sequences and ribosomal RNA reads were removed, and resulting reads were further cleaned for quality (Phred score 30) and minimum read length (80 bp) using the ea-utils tool (v1.1.2) fastq-mcf. The cleaned reads were normalised using Trinity’s (v2.2.0) *in silico* Read Normalisation tool (Haas, *et al.*, 2013), with default parameters. Normalised reads were *de novo* assembled using Trinity (v2.2.0) with default parameters (Grabherr, *et al.*, 2011). The longest isoform of each gene was extracted from the transcriptome for annotation as per An *et al.* (2014) using the Trinity utility script get_longest_isoform_seq_per (assembly statistics and assembled transcripts available for download in Supplementary File 1). The longest isoforms of each gene were compared to a local NCBI non-redundant (nr) database (updated 01 June 2016) using Basic Local Alignment Search Tool (blastx, cut-off <1e^-10^) to search for sequence similarity and putative gene annotation (Altschul, *et al.*, 1990).

Transcript abundance was estimated by alignment-based quantification using Trinity (v2.2.0) utilities (Grabherr, *et al.*, 2011; Haas, *et al.*, 2013). Transcripts were aligned to the *de novo* transcriptome using bowtie with default parameters and transcript abundance estimates were calculated using RNA-Seq by Expectation-Maximization (RSEM). A matrix of Trimmed Mean of M-values [TMM] normalised Fragments Per Kilobase Of Exon Per Million Fragments Mapped [FPKM] values was loaded into TM4 MultiExperiment Viewer (Howe, *et al.*, 2011) and clustered based on expression profiles for each gene using SOTA (Herrero, *et al.*, 2001).

ARACNe prediction of GRN

To construct a predicted GRN from the SOTA expression cluster profiles, ARACNe was implemented using “ARACNe for GNU/Linux” with a p-value cut-off of 1e^-7^ and DPI set to 0.1 (Margolin, *et al.*, 2006). The ARACNe network output (Supplementary file 2) was loaded in Cytoscape v3.4.0 for visualisation and exploration (Shannon, *et al.*, 2003). To identify highly interconnected sub-networks, GLay (clusterMaker) was applied (Supplementary information Fig.S1, (Morris, *et al.*, 2011)). Gene annotations (Blastx nr, as above) were mapped onto nodes in the regulatory gene network.

*In vivo* ground truth of biomineralisation modules

We have previously identified and characterised biomineralisation gene candidates in the Antarctic clam using a combination of semi-qPCR, *in situ* hybridisation (ISH) and proteomics (Sleight, *et al.*, 2016). Six of these candidate genes whose expression is specific to the calcifying outer epithelium of the mollusc mantle can be split into two groups. One set of translated proteins are also present in the nacreous shell proteome (*tyrA*, *chitin-binding domain, mytilin, pif*), whilst a second set do not have a translated protein detected in the nacreous shell proteome (*tyrB* and *Contig01043-unannotated transcript,* summarised in Fig. 2). These six candidate biomineralisation genes were used as molecular markers of biomineralisation, they were firstly all confirmed to be present in the current data set, and were then investigated in the ARACNe predicted GRN to test for predictions of co-expression and interaction via first and second neighbour connections.

Differential expression analysis

The gene-level abundance estimates (raw counts) for each of the libraries were constructed into a matrix for downstream expression analyses (using the Trinity abundance_estimates_to_matrix.pl script). Differentially expressed genes were identified using the Bioconductor (v3.4) edgeR package in R (v3.1.1) with a false discovery rate (FDR) of 5% (Robinson *et al.* 2010, McCarthy *et al.* 2012) and genes which showed significant temporal changes in response to damage, termed here, time-dependant damage-response genes, were identified. Genes that qualified as a time-dependent damage response gene in any of the three age categories were mapped onto the network. To investigate if the biomineralisation modules and priority candidates were enriched in time-dependent damage-response genes, all genes were ranked by FDR and the distributions of significant genes were assessed for different module conditions in each age category.

Enrichment analysis and identification of priority candidates using biomineralisation markers

The two modules that contained our previously defined biomineralisation markers were investigated. All of the trinity transcripts within each module were extracted and re-annotated using three databases: NCBI nr, Uniref90 and Swissprot human (Supplementary Files 3 & 4). Annotations were then analysed using StringDB and tested for enrichment against a whole genome background. In order to perform an *in silico* screen for priority candidate genes within the two biomineralisation modules two criteria were used: firstly, genes which had GO terms relating to functional categories with potential relevance to biomineralisation were identified (categories = receptors and signal transduction, ion transport, shell matrix proteins, biomineralisation enzymes and protein folding/chaperoning) and secondly, using literature searches, each gene was assessed to identify if it had previously been reported to play a role in the regulation of biomineralisation in other systems.

**Supplementary Results: Figures and Tables**

**Fig.S1. *Laternula elliptica* mantle tissue gene regulatory network created using ARACNE (p-value cut-off 1e^-7^) consisting of 13,577 nodes.** Highly interconnected regions identified using GLay yielding three large and fifteen small expression clusters, “large” clusters highlighted in red and “small” highlighted in green. The network file is available for download in Supplementary File 2.


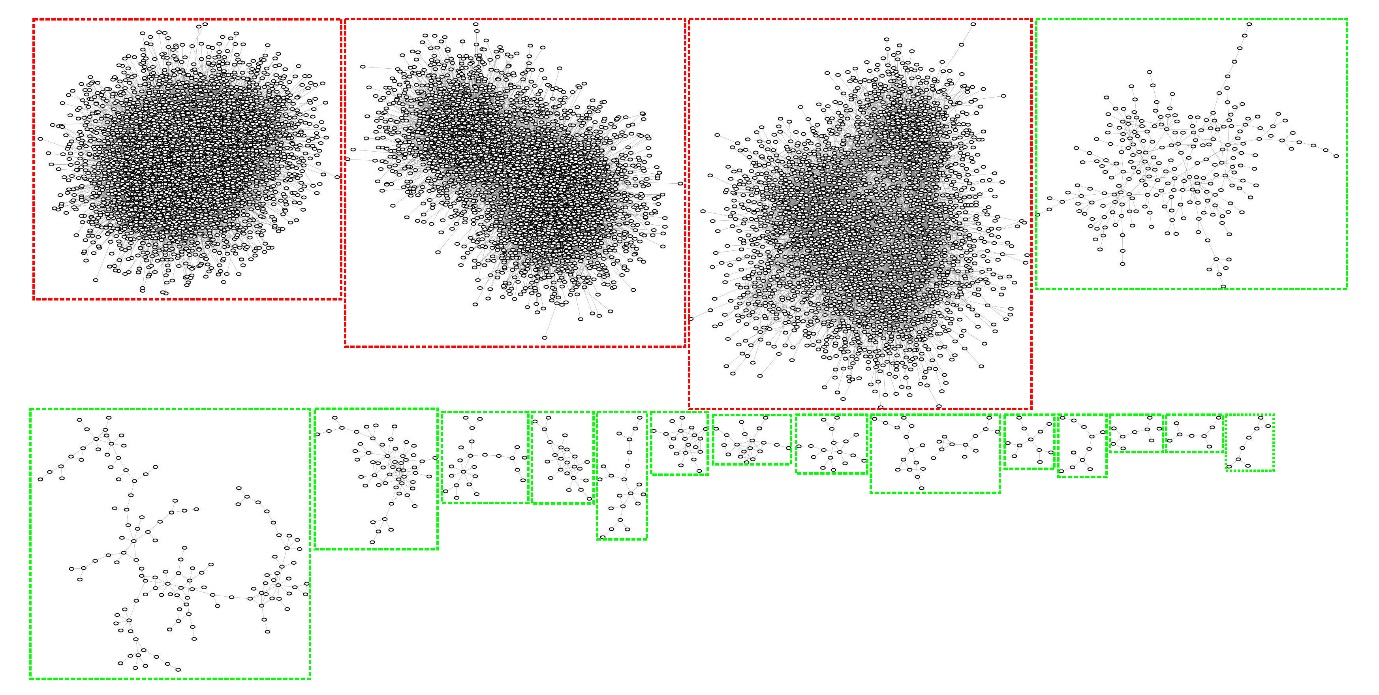
­­


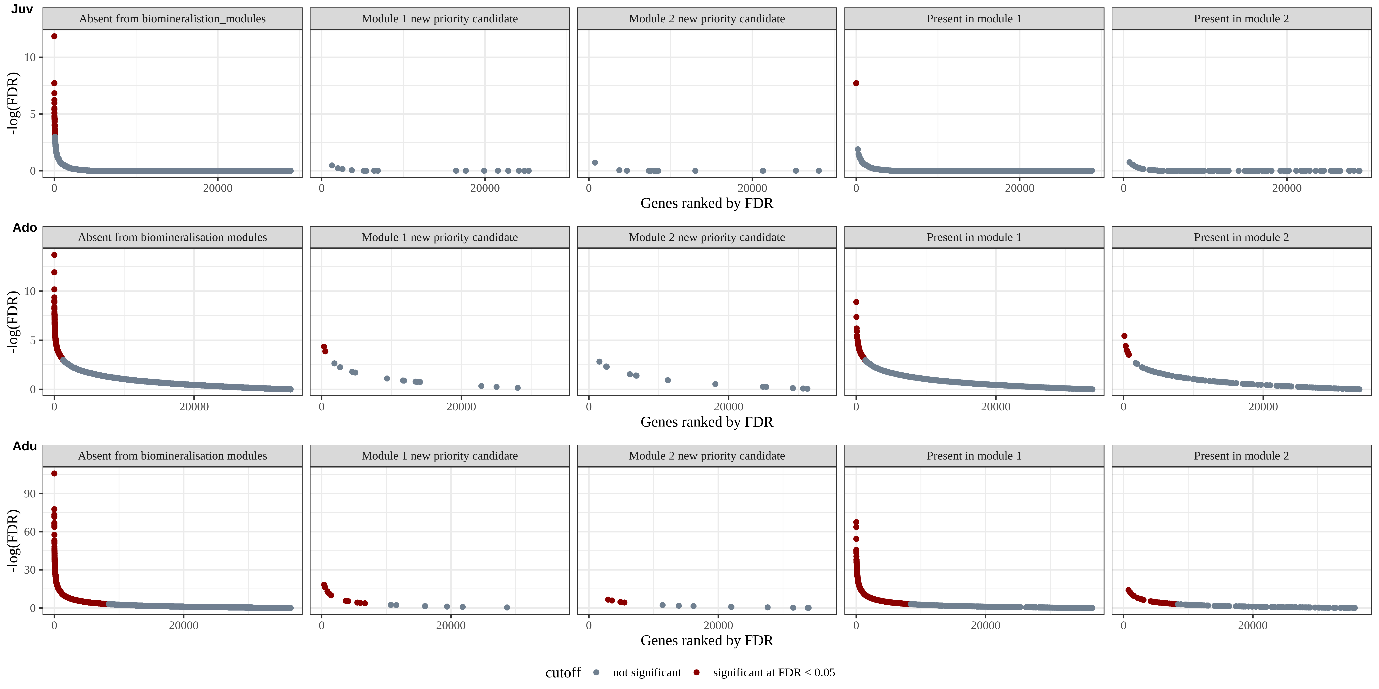


**Fig.S2. Assessment of time-dependent damage-response genes in each age category in the GRN.** Biomineralisation modules of the GRN and the priority gene lists do not contain a higher proportion of significant time-dependent damage-response genes (red).

| **Module** | **GRN Cluster ID** | **Trinity ID** | **Putative annotation** | **Functional group** |
| --- | --- | --- | --- | --- |
| **Module 1**  **Functional hypothesis =** *responsible for transcribing and translating the extracellular proteins required for biomineralisation of nacreous shell layer (see* *Supplementary information Table S1 and Fig.S3)* | Cluster-9690.txt | TRINITY_DN255896_c3_g2 | Toll-like receptor 4 | Receptor and signal transduction |
|  | Cluster-9690.txt | TRINITY_DN245288_c2_g1 | Toll-like receptor 2 |  |
|  | Cluster-2849.txt | TRINITY_DN246350_c2_g1 | Toll-like receptor 4 |  |
|  | Cluster-2128.txt | TRINITY_DN248655_c2_g5 | Neurogenic locus notch homolog protein 2 |  |
|  | Cluster-10028.txt | **TRINITY_DN251622_c0_g1** | Headcase protein homolog |  |
|  | Cluster-10469.txt | TRINITY_DN251675_c0_g1 | Forkhead box protein J1 |  |
|  | Cluster-2128.txt | TRINITY_DN253939_c2_g1 | Fibroblast growth factor receptor 3 |  |
|  | Cluster-18226.txt | TRINITY_DN242791_c2_g1 | Thioredoxin domain-containing protein 11 |  |
|  | Cluster-9587.txt | **TRINITY_DN256751_c1_g1** | Thioredoxin-related transmembrane protein 1 |  |
|  | Cluster-15239.txt | TRINITY_DN246576_c0_g1 | Akirin-1 |  |
|  | Cluster-2346.txt | **TRINITY_DN259824_c2_g1** | Solute carrier family 23 member 1 | Ion transport |
|  | Cluster-10050.txt | **TRINITY_DN256986_c3_g4** | Sodium/calcium exchanger 3 |  |
|  | Cluster-10335.txt | TRINITY_DN246390_c1_g1 | Pendrin |  |
|  | Cluster-13458.txt | **TRINITY_DN251420_c3_g1** | cartilage matrix protein-like | Shell matrix |
|  | Cluster-12543.txt | **TRINITY_DN242528_c0_g1** | Insoluble matrix shell protein 1 |  |
|  | Cluster-9662.txt | **TRINITY_DN250104_c5_g8** | PIF-like isoform X1 |  |
|  | Cluster-9665.txt | **TRINITY_DN244470_c1_g2** | PIF-like |  |
|  | Cluster-2079.txt | TRINITY_DN256420_c0_g2 | Carbonic anhydrase 2-like isoform X3 | Biomineralisation enzyme |
|  | Cluster-10478.txt | TRINITY_DN239234_c0_g1 | Heat shock 70 kDa protein 12B | Protein folding/chaperoning |
|  | Cluster-9701.txt | **TRINITY_DN249833_c1_g2** | Heat shock protein HSP 90-alpha |  |
|  | Cluster-16489.txt | **TRINITY_DN255921_c0_g1** | Heat shock factor-binding protein 1 |  |
| **Module 2**  **Functional hypothesis =** *responsible for the transport of both ions and proteins to biomineralisation site (see* *Supplementary information Table S2 and Fig.S4)* | Cluster-3198.txt | TRINITY_DN259845_c3_g3 | Alkaline phosphatase, tissue-nonspecific | Biomineralisation enzyme |
|  | Cluster-2772.txt | TRINITY_DN256081_c2_g5 | Tyrosinase |  |
|  | Cluster-71.txt | **TRINITY_DN253708_c3_g3** | Neurogenic locus notch homolog protein 2 | Receptor |
|  | Cluster-2764.txt | **TRINITY_DN248447_c0_g2** | Toll-like receptor 1 |  |
|  | Cluster-6852.txt | TRINITY_DN255895_c0_g3 | Protein transport protein Sec61 alpha-2 | Protein transport |
|  | Cluster-687.txt | TRINITY_DN244879_c4_g1 | Regucalcin | Ion transport |
|  | Cluster-6827.txt | TRINITY_DN237157_c0_g1 | Sodium/glucose cotransporter 4 |  |
|  | Cluster-2631.txt | **TRINITY_DN231473_c0_g2** | Sodium/nucleoside cotransporter 2 |  |
|  | Cluster-2568.txt | TRINITY_DN239292_c0_g1 | Sodium-coupled monocarboxylate transporter 2 |  |
|  | Cluster-606.txt | TRINITY_DN247229_c2_g1 | Sodium-dependent phosphate transport protein |  |
|  | Cluster-596.txt | TRINITY_DN253103_c2_g3 | Solute carrier family 22 member 4 |  |
|  | Cluster-10275.txt | **TRINITY_DN255933_c0_g1** | Solute carrier family 22 member 5 |  |
|  | Cluster-2690.txt | **TRINITY_DN245321_c4_g2** | Solute carrier family 23 member 1 |  |
|  | Cluster-2652.txt | TRINITY_DN250916_c0_g3 | Solute carrier family 28 member 3 |  |

**Table S1.** Hypothesised functional role and new priority annotated candidates from each of the biomineralisation modules in the computationally predicted mantle GRN, including Trinity ID and cluster mapping information.

_
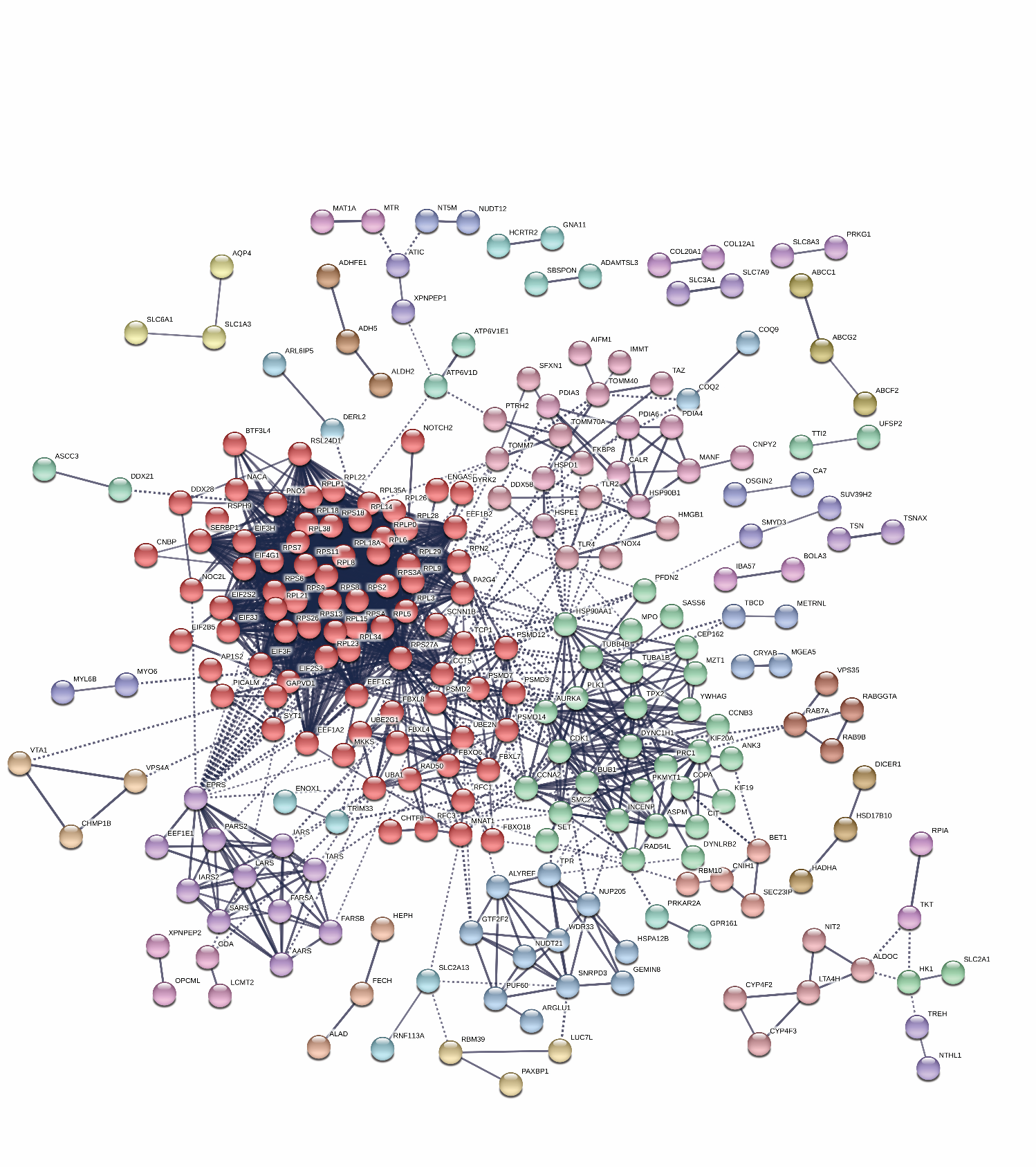
_

**Fig.S3. StringDB predicted protein interactions for all swissprot (human) annotated Trinity genes in GRN module 1 (Cluster-2730 1^st^ and 2^nd^ neighbours).** Node colours represent clustering (Markov Cluster Algorithm [MCL], inflation parameter = 2), weight of line indicates confidence of interaction, more detailed information on annotations can be found in corresponding supplementary file.

**Table S1.** Functional enrichment analysis, against whole genome background, of String DB network generated from module 1 (Cluster-2730 1^st^ and 2^nd^ neighbours).

| KEGG | | | |
| --- | --- | --- | --- |
| pathway ID | pathway description | observed gene count | false discovery rate |
| 3010 | Ribosome | 38 | 2.36E-31 |
| 970 | Aminoacyl-tRNA biosynthesis | 10 | 1.6E-06 |
| 3013 | RNA transport | 12 | 0.00588 |
| 4141 | Protein processing in endoplasmic reticulum | 12 | 0.00866 |
| 4145 | Phagosome | 11 | 0.00891 |
| 3050 | Proteasome | 6 | 0.0104 |
| Molecular Function (GO) – most significant 10 only | | | |
| pathway ID | pathway description | observed gene count | false discovery rate |
| GO.0003735 | structural constituent of ribosome | 32 | 9.37E-23 |
| GO.0003723 | RNA binding | 91 | 5.78E-19 |
| GO.0044822 | poly(A) RNA binding | 74 | 1.51E-16 |
| GO.0003824 | catalytic activity | 171 | 1.03E-12 |
| GO.0005198 | structural molecule activity | 43 | 2.92E-11 |
| GO.1901363 | heterocyclic compound binding | 167 | 3.13E-09 |
| GO.0097159 | organic cyclic compound binding | 167 | 8.30E-09 |
| GO.0036094 | small molecule binding | 98 | 1.94E-08 |
| GO.0000166 | nucleotide binding | 88 | 2.09E-07 |
| GO.0004812 | aminoacyl-tRNA ligase activity | 10 | 2.14E-06 |
| Biological Process (GO) – most significant 10 only | | | |
| pathway ID | pathway description | observed gene count | false discovery rate |
| GO.0006613 | cotranslational protein targeting to membrane | 39 | 2.74E-34 |
| GO.0045047 | protein targeting to ER | 39 | 2.74E-34 |
| GO.0019083 | viral transcription | 39 | 4.16E-34 |
| GO.0006614 | SRP-dependent cotranslational protein targeting to membrane | 38 | 8.28E-34 |
| GO.0070972 | protein localization to endoplasmic reticulum | 41 | 8.28E-34 |
| GO.0019080 | viral gene expression | 39 | 5.84E-33 |
| GO.0000184 | nuclear-transcribed mRNA catabolic process, nonsense-mediated decay | 38 | 2.04E-32 |
| GO.0006612 | protein targeting to membrane | 41 | 6.41E-30 |
| GO.0006413 | translational initiation | 44 | 9.12E-28 |
| GO.0006412 | translation | 50 | 1.79E-26 |
| Cellular Component (GO) – most significant 10 only | | | |
| pathway ID | pathway description | observed gene count | false discovery rate |
| GO.0022626 | cytosolic ribosome | 34 | 1.18E-31 |
| GO.0044445 | cytosolic part | 37 | 5.94E-25 |
| GO.0044391 | ribosomal subunit | 33 | 1.57E-24 |
| GO.0044444 | cytoplasmic part | 237 | 2.68E-22 |
| GO.0005829 | cytosol | 140 | 1.89E-20 |
| GO.0005840 | ribosome | 34 | 1.96E-19 |
| GO.0070062 | extracellular exosome | 122 | 4.75E-17 |
| GO.0022625 | cytosolic large ribosomal subunit | 18 | 9.31E-17 |
| GO.0022627 | cytosolic small ribosomal subunit | 16 | 1.33E-16 |
| GO.0005737 | cytoplasm | 272 | 4.09E-16 |

**Fig.S4. StringDB predicted protein interactions for all swissprot (human) annotated Trinity genes in GRN module 2 (Cluster-2775 1^st^ and 2^nd^ neighbours).** Node colours represent clustering (Markov Cluster Algorithm [MCL], inflation parameter = 2), weight of line indicates confidence of interaction, more detailed information on annotations can be found in corresponding supplementary file.


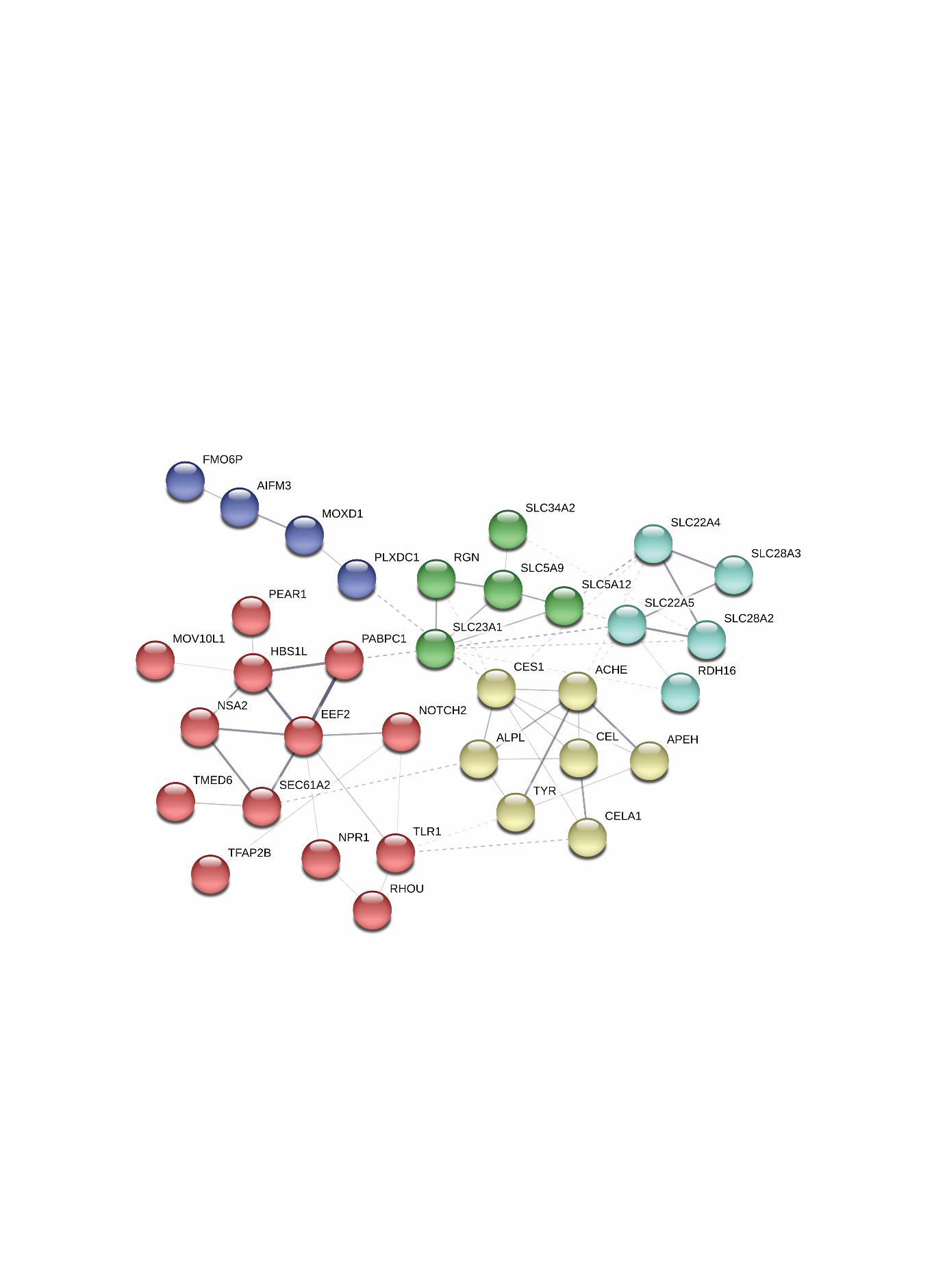


**Table S3.** Functional enrichment analysis, against whole genome background, of String DB network generated from module 2 annotations

| Biological Process (GO) | | | |
| --- | --- | --- | --- |
| pathway ID | pathway description | observed gene count | false discovery rate |
| GO.0006814 | sodium ion transport | 8 | 0.0000195 |
| Molecular Function (GO) | | | |
| pathway ID | pathway description | observed gene count | false discovery rate |
| GO.0015293 | symporter activity | 7 | 0.0000549 |
| GO.0005415 | nucleoside:sodium symporter activity | 2 | 0.00845 |
| GO.0015226 | carnitine transmembrane transporter activity | 2 | 0.00845 |
| GO.0008028 | monocarboxylic acid transmembrane transporter activity | 3 | 0.0205 |
| GO.0015077 | monovalent inorganic cation transmembrane transporter activity | 6 | 0.0205 |
| GO.0015205 | nucleobase transmembrane transporter activity | 2 | 0.0205 |
| GO.0015294 | solute:cation symporter activity | 4 | 0.0205 |
| GO.0046943 | carboxylic acid transmembrane transporter activity | 4 | 0.0205 |
| GO.1901618 | organic hydroxy compound transmembrane transporter activity | 3 | 0.0205 |
| GO.0008509 | anion transmembrane transporter activity | 5 | 0.0217 |
| GO.0052689 | carboxylic ester hydrolase activity | 4 | 0.0217 |
| GO.0015370 | solute:sodium symporter activity | 3 | 0.026 |
| GO.0015211 | purine nucleoside transmembrane transporter activity | 2 | 0.0261 |
| GO.0015081 | sodium ion transmembrane transporter activity | 4 | 0.0286 |
| GO.0016787 | hydrolase activity | 13 | 0.0443 |

**References**

Altschul,S.F. *et al.* (1990) Basic local alignment search tool. *J. Mol. Biol.,* **215**(3), 403-410.

An,J. *et al.* (2014) Transcriptome profiling to discover putative genes associated with paraquat resistance in Goosegrass (*Eleusine indica* L.). *Plos One*, **9**(6), e99940.

Grabherr,M.G. *et al.* (2011) Full-length transcriptome assembly from RNA-Seq data without a reference genome. *Nature Biotechnology,* **29**(7), 644-U130.

Grabherr,M.G. *et al.* (2011) Full-length transcriptome assembly from RNA-Seq data without a reference genome. *Nature Biotechnology,* **29**(7), 644-U130.

Howe,E.A. *et al.* (2011) RNA-Seq analysis in MeV. *Bioinformatics*, **27**(22), 3209-3210.

McCarthy,D.J. *et al.* (2012) Differential expression analysis of multifactor RNA-Seq experiments with respect to biological variation. *Nucleic Acids Res.,* **40**(10), 4288-4297.

Morris,J.H. *et al.* (2011) clusterMaker: a multi-algorithm clustering plugin for Cytoscape. *BMC Bioinformatics,* **12**, 436.

Robinson,M.D. *et al.* (2010) edgeR: a Bioconductor package for differential expression analysis of digital gene expression data. *Bioinformatics*, **26**(1), 139-140.

Shannon,P. *et al.* (2003) Cytoscape: A software environment for integrated models of biomolecular interaction networks. *Genome Research*, **13**(11), 2498-2504.

Sleight,V.A. *et al.* (2016) An Antarctic molluscan biomineralisation tool-kit. *Sci. Rep.,* **6**, 36978.
